# Hypodermosis by *Hypoderma diana* (Diptera: Hypodermatinae) in the Manchurian Wapiti (*Cervus canadensis xanthopygus*)

**DOI:** 10.3390/ani16101461

**Published:** 2026-05-10

**Authors:** Xingkun Yang, Qinglin Wu, Xuejun Zhang, Yinduleng Sai, Yvanyvan Ma, Shumiao Zhang, Wanda Li, Thomas Pape, Dong Zhang

**Affiliations:** 1 School of Nature Conservation, Beijing Forestry University, Beijing 100083, China; xingkun_yang@bjfu.edu.cn (X.Y.); myy486527917@163.com (Y.M.); 2 Inner Mongolia Gaogesitai Hanwula National Nature Reserve Administration, Tianshan 025550, China; qinglin_wu@yeah.net (Q.W.); zhangxuejun1012@163.com (X.Z.); saiyinduleng@126.com (Y.S.); 3 Beijing Milu Ecological Research Center, Beijing 100076, China; shumiaozhang@126.com; 4 Beijing Zoo, Beijing 100044, China; liwanda1@163.com; 5 Natural History Museum of Denmark, Science Faculty, University of Copenhagen, 2100 Copenhagen, Denmark; tpape@snm.ku.dk

**Keywords:** antennomaxillary sensory complex, *Hypoderma diana*, hypodermosis, mitochondrial COI, myiasis, scanning electron microscopy

## Abstract

*Hypoderma diana* Brauer, 1858, poses a significant threat to species of deer, yet records of this species in China remain fragmented and lack comprehensive documentation. Following high mortality in wapiti populations in Inner Mongolia during 2023–2024, surveys were conducted in the Gaogesitai Hanwula Nature Reserve in March 2025. Species identity was established through rigorous morphological analysis of third-instar larvae and an emerged adult female using light and scanning electron microscopy, supplemented by a 689 bp COI fragment. Results confirmed *H. diana* as the causative agent, with larvae localized along the host’s vertebral column. Comparative SEM analysis of the antennomaxillary sensory complex in *H. diana*, *H. bovis* (Linnaeus, 1758), and *H. lineatum* (Villers, 1789) revealed undifferentiated, volcano-like warts, contrasting sharply with the highly differentiated antennae and sensilla of *Oestrus ovis* Linnaeus, 1758. This study represents an inaugural record of *H. diana* in the Chifeng region and provides the first atlas of diagnostic features for both third-instar larvae and adults. These findings document *H. diana* as a critical agent of myiasis in the Manchurian wapiti, refining its known epidemiological profile in China and establishing an essential morphological reference for future wildlife health surveillance.

## Simple Summary

Warble flies are parasites whose larvae, or maggots, live under the skin of animals, causing a disease that can lead to high death rates in wild deer populations. In China, records of these flies are very limited, and a lack of clear photographs makes it difficult for researchers and park staff to identify them. This study examined a disease outbreak in the Manchurian Wapiti, a type of large deer, in Inner Mongolia to better understand where these flies live and what they look like. We successfully identified the fly species and produced the first detailed photographs of both the adult female fly and its larvae in China. We also discovered a new physical marker on the head of the larvae and recorded its molecular barcode to help future identification efforts. By recording this parasite for the first time in the Chifeng region and providing a visual guide, our work helps wildlife managers to monitor animal health. This research is vital for protecting deer populations and managing parasitic diseases in nature.

## 1. Introduction

Warble flies (Oestridae, Hypodermatinae) have garnered significant scientific interest due to their larvae causing myiasis in a broad range of mammals [1,2,3,4]. The genus *Hypoderma* Latreille contains most of the ruminant-parasitising species of subfamily Hypodermatinae [2,5]. The larvae are obligate parasites in ruminants, with the first instar migrating through subcutaneous connective tissue or along nerve pathways and fascial planes between muscles, and the second and third instars settling in subcutaneous swellings (“warbles”), usually in the dorsolumbar region [1,2]. Severe infections lead to hypodermosis with substantial economic impacts for livestock production [1,5]. Species of *Hypoderma* generally exhibit a high degree of host specificity, although they frequently coexsist in the same ecological environments and often share hosts: *H. tarandi* (Linnaeus, 1758) primarily parasitizes reindeer (*Rangifer tarandus*), *H. bovis* (Linnaeus, 1758) and *H. lineatum* (Villers, 1789) predominantly infect cattle, and *H. actaeon* Brauer, 1858 is primarily adapted to the red deer (*Cervus elaphus* Linnaeus, 1758) [6,7], although incidental parasitism has also been documented in other cervids, including the roe deer (*Capreolus capreolus* Linnaeus, 1758) [8] and the fallow deer (*Dama dama* (Linnaeus, 1758)) [9]. In contrast, *H. diana* has a broader host spectrum, primarily infecting roe deer and red deer (*Cervus elaphus*) [1], as well as several other species of deer [1]. Sporadic cases of *H. diana* infestation have also been reported in non-cervid hosts, including horses [10,11], alpacas (*Vicugna pacos* (Linnaeus, 1758)) [12], lechwe (*Kobus lechwe* Gray, 1850) [1], sheep (*Ovis aries* Linnaeus, 1758) [13], and in a single case, a wild boar (*Sus scrofa* Linnaeus, 1758) [14]. Human hypodermosis is rare and largely sporadic, predominantly occurring in infants, with documented cases attributed to *H. bovis*, *H. tarandi*, and *H. diana* [6,7,15,16]. These findings highlight the dual burden of hypodermosis, encompassing both significant economic losses and public health concerns [17,18,19,20].

Females of *H. diana* typically glue eggs to hairs on the host’s hindquarters, back, thighs, flanks, and abdomen. After hatching, the larvae enter the host and migrate subcutaneously towards the spinal column and eventually settle along the host’s back [1]. The distribution of *H. diana* extends from Europe to the Russian Far East [1], with its primary range in China concentrated in the northern and northwestern regions [10]. The primary host of *H. diana* in China is the Manchurian wapiti, *Cervus canadensis xanthopygus* (H. Milne-Edwards, 1867) [21], which is a second-class protected species [22] and remains poorly studied [23].

Third-instar larvae of *H. diana* are only reported in specific hosts like roe deer and red deer, confirming the ability of these hosts to support complete development [23]. In these hosts, larvae are localized in the dorsal subcutaneous tissues, concentrated along the spinal column from the shoulders to 10 cm anterior to the tail. Infection intensity varies, and yearling red deer may harbour over 500 larvae, while 2.5-year-old animals may carry as few as four larvae or, in some instances, be entirely free of larvae. Roe deer may show an average infection load of 150–180 larvae per individual. In non-specific hosts, only first- and second-instar larvae have been found, indicating incomplete development [10,12].

Ruminant-parasitising species of Hypodermatinae pose a parasitological challenge in veterinary medicine, yet their distribution and impact remain poorly documented in wildlife, primarily due to the complexities of obtaining wildlife samples [6]. Challenges for larval studies include the low infestation rates in wild hosts and the difficulties in handling large hosts for in situ studies. More recently, widespread Ivermectin use in livestock has drastically reduced larval populations [2], reducing access to larvae in domestic ruminants. The main obstacles for research on adult Hypodermatinae are the extremely short adult lifespan and the sparse knowledge about mating sites and similar data on their natural occurrence [1].

## 2. Materials and Methods

### 2.1. Study Area and Specimen Collection

Field investigations were conducted in the Inner Mongolia Gaogesitai Hanwula National Nature Reserve (44°41′03″–45°08′44″ N, 119°03′30″–119°39′08″ E) over two periods: from 12 March to 18 March 2025 and on 22 March 2025. During these surveys, we identified six deer carcasses, including two affected by hypodermosis, and one critically ill red deer also affected by hypodermosis. To facilitate specimen collection, the dermis was carefully reflected from the underlying musculature via a longitudinal incision along the vertebral column. Third-instar larvae were then manually extracted from the exposed subcutaneous pockets and preserved in 75% ethanol for morphological studies and in 100% ethanol for molecular analysis. Additionally, twelve puparia were collected from the critically ill red deer. These puparia were then placed on a substrate of tissue paper and maintained in the laboratory at a constant temperature of 25 °C to facilitate adult emergence. This protocol resulted in the successful emergence of a single female adult. All sampling procedures were conducted with the support of a local veterinarian and received approval from the Inner Mongolia Gaogesitai Hanwula National Nature Reserve Administration as well as the School of Nature Conservation at Beijing Forestry University. Voucher specimens are deposited at Beijing Forestry University.

Comparative specimens of *H. bovis*, *H. lineatum*, and *Oestrus ovis* were obtained from the long-term repository of Beijing Forestry University, where they have been maintained in 75% ethanol for long-term preservation.

### 2.2. Morphological Identification and Terminology

For scanning electron microscopy (SEM), specimen cleaning involved 10 min at 60 Hz in a dedicated cleaning solution (KQ5200E, Kun Shan Ultrasonic Instruments, Kun shan, China. Subsequently, the larvae were washed three times in phosphate-buffered saline (PBS; Solarbio, Beijing, China) at room temperature to provide a clean surface for further analysis. Dehydration was achieved through immersion in ethanol, progressing through concentrations of 70%, 80%, 90%, 95%, and 100% ethanol, with each concentration maintained for 30 min. To capture a frontal view of the pseudocephalon and posterior spiracles, one larva was carefully sectioned between the second and third thoracic segments and between the sixth and seventh abdominal segments using a fine blade, preserving only the regions containing the pseudocephalon and posterior spiracles. The specimens were then dried using CO_2_ and mounted on stubs with colloidal silver paint. Subsequently, the specimens were sputter-coated with gold using an ion sputter coater and analyzed using a field-emission scanning electron microscope (Hitachi SU8010) (HITACHI, Tokyo, Japan). For light microscopy (LM), third-instar larvae and the adult female were photographed using a ZEISS SteREO Discovery V2.0 (Carl Zeiss Microscopy GmbH, Jena, Germany).

Specimen identification was based on the works of Zumpt [1] and Colwell et al. [24] for larvae, and Xue et al. [25] and Grunin [26] for adults. Morphological terms follow the standards outlined in these references.

### 2.3. Molecular Identification

DNA extraction was performed using the TIANamp Micro DNA Kit (Tiangen, China) in accordance with the manufacturer’s protocol, and the extracted DNA was stored at −20 °C. Mitochondrial COI sequences were amplified using universal primers (LCO and HCO) [27]. The PCR reaction mixture consisted of 8.5 μL DNA template, 2 μL BSA, 1 μL of each bidirectional primer, and 12.5 μL of 2 × Es Taq MasterMix (Cowin Bioscience, China), with a total reaction volume of 20 μL. The PCR protocol included an initial denaturation at 94 °C for 3 min, followed by 35 cycles of denaturation at 94 °C for 30 s, annealing at 54 °C for 30 s, and extension at 72 °C for 45 s, with a final extension at 72 °C for 10 min. Post-amplification, 3 μL of the PCR product was subjected to electrophoresis on a 1% agarose gel stained with GoldView. Positive PCR products were purified and sent to the Beijing Genomics Institute (BGI, China) for bidirectional sequencing.

## 3. Results

### 3.1. Morphology of Third-Instar Larvae of Hypoderma diana

**Figure 1:**
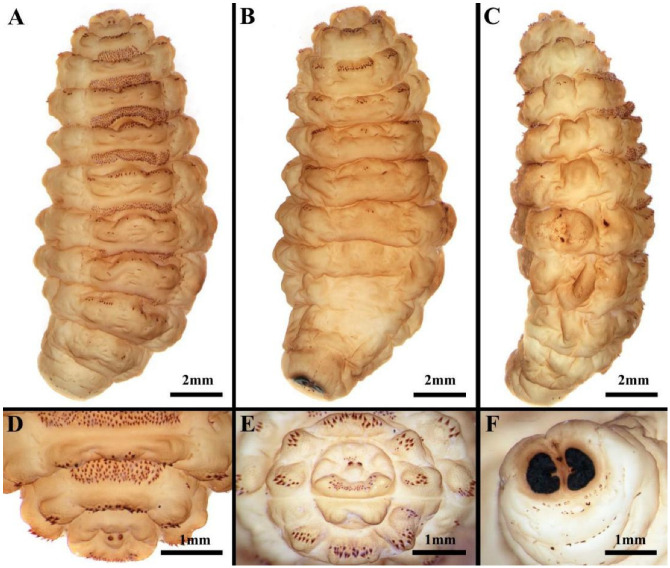
*Hypoderma diana*, third-instar larvae. (**A**) Habitus, ventral view. (**B**) Habitus, dorsal view. (**C**) Habitus, lateral view. (**D**) Pseudocephalon and first two thoracic segments, ventral view. (**E**) Pseudocephalon, anterior view. (**F**) Anal division, posterior view.

**Figure 2:**
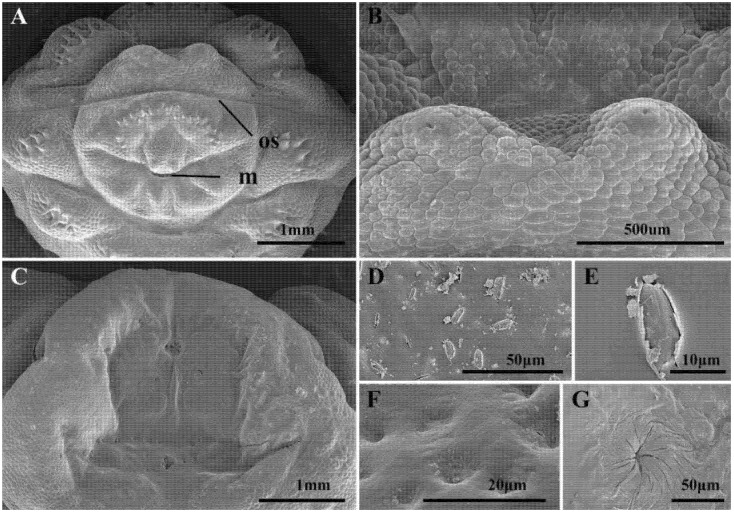
*Hypoderma diana* third-instar larva scanning electron microscopy details. (**A**) Pseudocephalon and first thoracic segment, anterior view. (**B**) Pair of median papillae presented dorsally on pseudocephalon. (**C**) Anal division, caudal view showing posterior spiracular plate. (**D**) Slitlike spiracular openings on surface of spiracular plate. (**E**) Enlargement of slitlike spiracular opening. (**F**) Depression in the spiracular plate. (**G**) Ecdysial scar. Abbreviations: m, mouth opening; os, opercular suture.

The third-instar larvae of *H. diana* measure 2.0–2.5 cm in length, are fusiform, milky white to beige, and divided into the 12 body parts typical for calyptrate maggots: pseudocephalon, three thoracic segments, seven abdominal segments and the anal division carrying the posterior spiracles (Figure 1A–F and Figure 2A,C).

Spines are present on the pseudocephalon in two bands: a cluster of spines is present between the mouth (m) and the opercular suture (os) (Figure 2A). The spines at the posterior margin of both the dorsal and ventral surfaces of the first thoracic segment are narrow and taper gradually to sharp points. The ventral spines are densely packed, with double spinulation extending from the first abdominal segment to abdominal segment six, while only an anterior row of spines is present on abdominal segment seven, presenting small hook-like spines with a stout base (Figure 1A–C). Multiple zygomorphic warts are present on each thoracic and abdominal segment, with each having a sensory pit at the top, and the general cuticle is covered in small, scale-like sclerotisations (Figure 2B).

Posterior spiracles with each posterior spiracular plate forming an irregularly edged oval (Figure 1F and Figure 2C). The ecdysial scar is positioned near the middle of each plate, closer to the median, and it is surrounded by spirally folded cuticle (Figure 2G). The spiracular plates exhibit a prominent ‘C’-shaped structure, which is generally flat and does not completely envelop the ecdysal scar (Figure 2C,F). Each plate contains numerous slit-like openings, each encircled by a slightly raised cuticular rim that lacks ornamentation (Figure 2D,E).

Scanning electron microscopy of the antennomaxillary sensory complex in *H. diana*, *H. lineatum*, and *H. bovis* revealed that this structure is not markedly differentiated, appearing instead as volcano-like warts. This stands in stark contrast to the larvae of *Oestrus ovis*, which exhibit distinct morphological differentiation of both the antennae and their associated maxillary sensilla.

**Figure 3:**
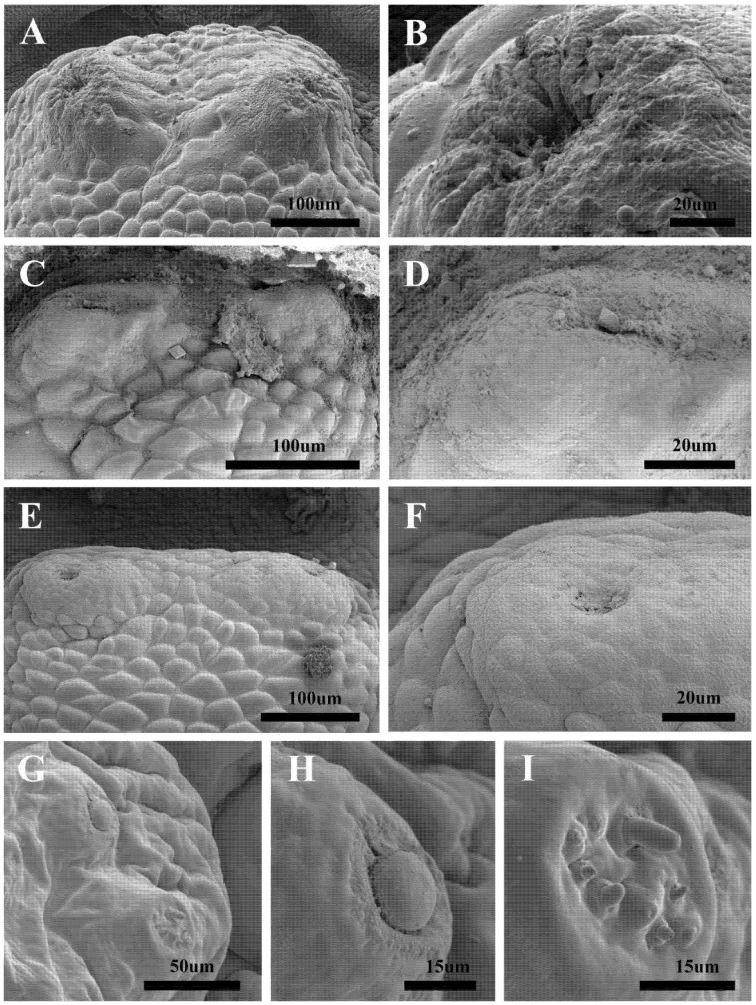
Scanning electron microscopy of the antennomaxillary sensory complex in selected third-instar Oestridae larvae. (**A**,**B**) Antennomaxillary sensory complex of *Hypoderma diana*. (**C**,**D**) Antennomaxillary sensory complex of *Hypoderma bovis.* (**E**,**F**) Antennomaxillary sensory complex of *Hypoderma lineatum*. (**G**–**I**) Antennomaxillary sensory complex, including antennal dome and maxillary palpus of *Oestrus ovis*.

These volcano-like warts are characterized by a prominent apical depression; notably, the central part of these cavities is remarkably uniform, with no discernible differentiation of specialized sensilla observed within the depressed region (Figure 3A–F). In stark contrast, the antennomaxillary sensory complex in *O. ovis* is markedly differentiated, comprising a distinct antennal dome and a maxillary palpus, the latter of which incorporates a diverse array of specialized sensilla (Figure 3G–I).

### 3.2. Morphology of Adult Female Hypoderma diana

**Figure 4:**
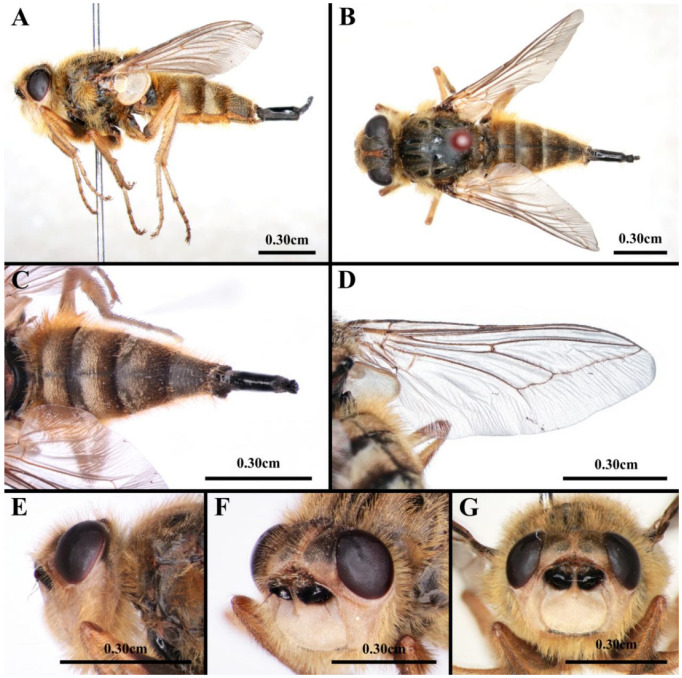
*Hypoderma diana*, adult female. (**A**) Habitus, lateral view. (**B**) Habitus, dorsal view. (**C**) Abdomen, dorsal view. (**D**) Wing, dorsal view. (**E**) Head, lateral view. (**F**) Head, anterolateral view. (**G**) Head, anterior view.

Body length 15.7 mm (Figure 4A,B). Eyes bare, dark brown. Anterior half of fronto-orbital plate and parafacial yellow with yellow soft setae; posterior half of fronto-orbital plate and postocular strip with black soft setae; occiput, gena and clypeus densely covered with long yellow soft setae (Figure 4E–G). Thorax ground color black with sparse yellowish-brown pollinosity and dense, short yellowish-brown soft setae. Scutellum shiny black with distinct central indentation at posterior margin (Figure 4B). Abdomen long oval, syntergite 1 + 2 to tergite 5 densely clothed with yellowish-brown soft setae, sternites 1–5 with dense, long, soft setae, sternite 2 tongue-shaped with rounded posterior end (Figure 4C). Terminalia shiny black with pointed tip (Figure 4A,C). Wing hyaline, with dark yellow venation; basicosta black, bare; tegula brown; costal spine not differentiated; vein R1 bare; crossvein r-m located distal to the end of subcostal vein; no setae dorsally at base of vein m_3+4_ (Figure 4D), lower calypter dingy white with brown outer margin; halter dark brown (Figure 4A). Legs, mostly yellowish-brown, proximal parts of mid-leg and hind femur black, basal half of fore femur black. Femur ventrally with golden, soft setae. Claws black, pulvillus dingy white. Fore femur with one row of long black setae ventrally in distal half; distal part of mid-femur and base of mid-tibia slender; hind femur with 2–3 ventral setae distally (Figure 4A).

### 3.3. Molecular Evidence

In the present study, a 689 bp fragment of the mitochondrial cytochrome c oxidase subunit I (COI) gene was successfully amplified and sequenced. A direct match with the existing GenBank sequence (Accession No. AF497763.1) was not achieved, and alignment through Geneious Prime 2023.2.1 (Biomatters, Auckland, New Zealand) confirmed that this discrepancy is due to the two sequences targeting different, non-overlapping regions of the COI gene. Consequently, these new data significantly supplement the molecular library for *H. diana* and provide robust molecular support for its taxonomic identification.

### 3.4. Case Observations

According to reports provided by the staff of the Inner Mongolia Gaogesitai Hanwula National Nature Reserve, field investigations into *Hypoderma diana* infestations in red deer revealed a pronounced increase in mortality rates during the severe winter (November 2023 to February 2024; mean monthly temperature: −13.5 °C) and the subsequent spring (March to May 2024; mean monthly temperature: 6.8 °C). These communications highlighted a disproportionate impact on females and juvenile individuals under three years of age. Infected animals displayed a range of clinical manifestations, including restlessness, unkempt pelage, spinal cysts, and limb parasitism, which significantly compromised their locomotor capabilities.

**Figure 5:**
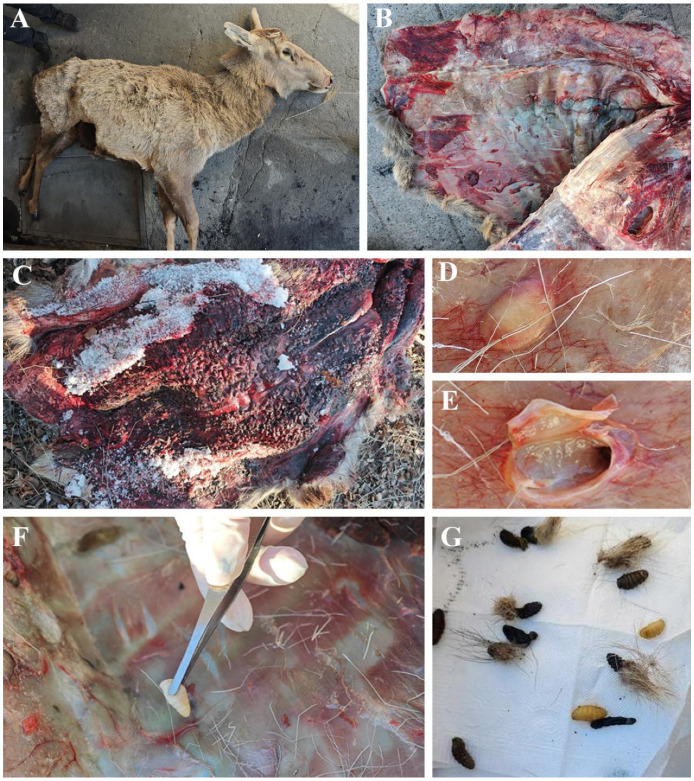
*Hypoderma diana*, larval infection. (**A**) Moribund host. (**B**,**C**) *H. diana* larval subcutaneous site of infection. (**D**,**E**) Abscess at the site of infection and internal morphology. (**F**) Collection process of *H. diana* larvae. (**G**) Pupariating larvae.

Post-mortem examinations conducted during the study identified four dead third-instar larvae within the dermal layer adjacent to the vertebral canal of a 2.5-year-old male (Figure 5B). Furthermore, a 1-year-old male, who had sustained a leg fracture, presumably attributable to harsh winter conditions, harboured over 500 larvae distributed within 15–20 cm from the spinal column (Figure 5A,C). Notably, a moribund 3-year-old male exhibited dermal swellings, accompanied by larvae undergoing pupariation (Figure 5G), underscoring the profound pathological consequences of *H. diana* infestations on red deer populations.

## 4. Discussion

Colwell et al. [24] and Otranto et al. [28] utilized scanning electron microscopy to delineate the morphological distinctions among third-instar larvae of *Hypoderma tarandi*, *H. bovis*, *H. lineatum*, *H. diana*, and *H. actaeon*. Our findings corroborate these studies, confirming the diagnostic value of traditionally recognized features. However, our investigation introduces the ‘volcano-like’ antennomaxillary sensory complex as a novel character state diagnostic for part or all of the genus *Hypoderma*. Our SEM analysis reveals that in the three *Hypoderma* species examined, the antennomaxillary sensory complex lacks marked differentiation and appears instead as a volcano-like wart. This structural simplicity stands in stark contrast to third-instar larvae of the other bot fly subfamilies, which exhibit distinctly differentiated antennae and maxillary sensory organs [2,29].

Regarding adult morphology, Zumpt [1] and Nilssen et al. [30] documented that *H. tarandi*, *H. bovis*, and *H. lineatum* exhibit a bumblebee-like appearance, a trait hypothesized to enhance their survival through mimicry. Conversely, *H. diana* and *H. actaeon* lack such mimicry and instead possess colour patterns that may facilitate mate recognition. Notably, *H. diana* is characterized by the presence of yellow setae on the epistome, while *H. actaeon* displays white setae in the same region. Furthermore, *H. qinghaiense* (Fan, 1982) is distinguished by black setae on the parafrontalia and parafacialia [31].

Parasitic infections significantly modulate wildlife population dynamics [32,33,34]. Within China, biogeographical range of *Hypoderma diana* has historically remained remarkably poorly documented, with fragmented records confined to Heilongjiang, Xinjiang, and the Hulunbuir region of Inner Mongolia [25]. A critical lacuna in previous domestic literature is the absence of verified photographic documentation, which has historically hindered reliable morphological identification and field surveillance. This investigation addresses these gaps by establishing the first inaugural record of *H. diana* from Chifeng, Inner Mongolia, while simultaneously providing the first domestic diagnostic atlas of both the third-instar larvae and the female adults. These findings substantially refine the known epidemiological profile of *H. diana* in China and offer an indispensable morphological reference for future research into the biological factors governing its relationship with cervid hosts.

## 5. Conclusions

This study establishes the first record of *Hypoderma diana* in the Chifeng region of Inner Mongolia. The investigation provides several key contributions to the field: it presents the first domestic diagnostic atlas for both the third-instar larvae and the female adult, offers a detailed morphological characterization of the adult female, and identifies the volcano-like antennomaxillary sensory complex as a novel diagnostic marker in larvae of *Hypoderma* spp. Additionally, a 689 bp COI fragment was successfully sequenced to supplement existing molecular databases. Collectively, these findings significantly refine the known distributional and epidemiological profile of *H. diana* in China. This work establishes a robust morphological and molecular baseline, providing an essential reference for future research into host–parasite dynamics and the health surveillance of wild cervid populations.

## Data Availability

The data generated by this study are provided here, and they are also available upon request from the corresponding author.
